# Cryopreservation Preserves Cell-Type Composition and Gene Expression Profiles in Bone Marrow Aspirates From Multiple Myeloma Patients

**DOI:** 10.3389/fgene.2021.663487

**Published:** 2021-04-21

**Authors:** Duojiao Chen, Mohammad I. Abu Zaid, Jill L. Reiter, Magdalena Czader, Lin Wang, Patrick McGuire, Xiaoling Xuei, Hongyu Gao, Kun Huang, Rafat Abonour, Brian A. Walker, Yunlong Liu

**Affiliations:** ^1^Department of Medical and Molecular Genetics, Indiana University School of Medicine, Indianapolis, IN, United States; ^2^Center for Computational Biology and Bioinformatics, Indiana University School of Medicine, Indianapolis, IN, United States; ^3^Department of BioHealth Informatics, School of Informatics and Computing, Indiana University-Purdue University Indianapolis, Indianapolis, IN, United States; ^4^Division of Hematology and Oncology, Department of Medicine, Indiana University School of Medicine, Indianapolis, IN, United States; ^5^Bone Marrow and Blood Stem Cell Transplantation Program, Indiana University Health, Indianapolis, IN, United States; ^6^Department of Pathology, Indiana University School of Medicine, Indianapolis, IN, United States; ^7^Center for Medical Genomics, Indiana University School of Medicine, Indianapolis, IN, United States

**Keywords:** cryopreservation, multiple myeloma, single-cell RNA sequencing, DMSO, bone marrow aspirate

## Abstract

Single-cell RNA sequencing reveals gene expression differences between individual cells and also identifies different cell populations that are present in the bulk starting material. To obtain an accurate assessment of patient samples, single-cell suspensions need to be generated as soon as possible once the tissue or sample has been collected. However, this requirement poses logistical challenges for experimental designs involving multiple samples from the same subject since these samples would ideally be processed at the same time to minimize technical variation in data analysis. Although cryopreservation has been shown to largely preserve the transcriptome, it is unclear whether the freeze-thaw process might alter gene expression profiles in a cell-type specific manner or whether changes in cell-type proportions might also occur. To address these questions in the context of multiple myeloma clinical studies, we performed single-cell RNA sequencing (scRNA-seq) to compare fresh and frozen cells isolated from bone marrow aspirates of six multiple myeloma patients, analyzing both myeloma cells (CD138^+^) and cells constituting the microenvironment (CD138−). We found that cryopreservation using 90% fetal calf serum and 10% dimethyl sulfoxide resulted in highly consistent gene expression profiles when comparing fresh and frozen samples from the same patient for both CD138^+^ myeloma cells (*R* ≥ 0.96) and for CD138^–^ cells (*R* ≥ 0.9). We also demonstrate that CD138^–^ cell-type proportions showed minimal alterations, which were mainly related to small differences in immune cell subtype sensitivity to the freeze-thaw procedures. Therefore, when processing fresh multiple myeloma samples is not feasible, cryopreservation is a useful option in single-cell profiling studies.

## Introduction

Single-cell RNA sequencing (scRNA-seq) allows transcriptome analysis at the single-cell level and has rapidly become one of the most popular techniques in biomedical research (Lafzi et al., 2018). This high-throughput technology makes it possible to measure cell-type specific gene expression in tens of thousands of cells in a single experiment (Chen et al., 2018). Not only has scRNA-seq increased the resolution of transcriptome analysis, but it also can identify low abundance cell populations. As a result, scRNA-seq has allowed researchers to map the contributions of different cell types in disease and this approach is now widely used in cancer clinical trials to monitor disease progression and response to therapy.

High-quality single-cell suspensions are required for scRNA-seq experiments (Lafzi et al., 2018). Once tissue biopsies have been removed from a patient, they begin to undergo ischemia-related gene expression changes and RNA degradation (Grizzle et al., 2016; Guo et al., 2020). To obtain an accurate profile of the patient sample, these types of changes should be minimized by processing samples as soon as they have been collected (Grizzle et al., 2016; Guo et al., 2020). In addition, if cell-type specific gene expression differences are to be compared in samples collected from the same subject, these samples ideally would be processed at the same time to minimize experimental batch effects (Alles et al., 2017). However, these best practices can pose logistical challenges. For instance, if a study requires knowledge of the clinical outcome before selecting samples for single-cell analysis, the experiment cannot be conducted at the time of sample collection. In addition, longitudinal studies often require the collection of samples over a time period of several weeks or longer. Therefore, in such scenarios a more feasible solution would be to cryopreserve single-cell suspensions of freshly isolated samples for future analysis (Alles et al., 2017; Guillaumet-Adkins et al., 2017; Attar et al., 2018; Hentze et al., 2019).

Viable frozen cells can be cryopreserved using a freezing mix of dimethyl sulfoxide (DMSO) with media or serum and stored for many years in liquid nitrogen for use in subsequent experiments (Tedder et al., 1995; Cox et al., 2005; Weinberg et al., 2010; Silveira et al., 2013). Although cryopreservation using DMSO has been shown to be more robust than other freezing protocols (Zheng et al., 2017; Wohnhaas et al., 2019), the influence of the freezing and thawing process on the transcriptome of different cell types is still a primary concern. While DMSO cryopreservation has been reported to have minimal effects on gene expression profiles in thawed cells, it has been noted to alter cell-type proportions (Zheng et al., 2017; Bahsoun et al., 2019; Wohnhaas et al., 2019). For example, scRNA-seq experiments using human peripheral blood mononuclear cells (PBMC) and monocyte-derived macrophages indicated that while cell populations and gene-expression signatures found in the fresh samples were largely preserved in frozen samples after thawing, differences were observed in the relative abundance of NK cells, plasmacytoid dendritic cells and macrophages (Zheng et al., 2017; Bahsoun et al., 2019; Wohnhaas et al., 2019). Therefore, the effect of cryopreservation on gene expression profiles and cell-type proportions should be determined empirically before use in clinical studies.

In this study, we evaluated the impact of cryopreservation on cells isolated from bone marrow aspirates from patients with multiple myeloma using scRNA-seq. Myeloma is a B-cell neoplasia characterized by the clonal expansion of malignant plasma cells in the bone marrow (Chesi and Bergsagel, 2011). The progression of normal plasma cells to myeloma cells involves two major genetic events: hyperdiploidy and translocations involving the *IgH* locus on chromosome 14 (Saxe et al., 2019). Like other tumors, multiple myeloma exhibits a complex ecosystem where tumor cells release extracellular factors that signal cells in the surrounding bone marrow microenvironment to promote angiogenesis, immune tolerance, and tumor growth (Caligaris-Cappio et al., 1992; Manier et al., 2012; Kawano et al., 2015). Thus, we also examined the effect of cryopreservation on both the tumor cells and their microenvironment in samples obtained from the same patient.

## Results

**FIGURE 1:**
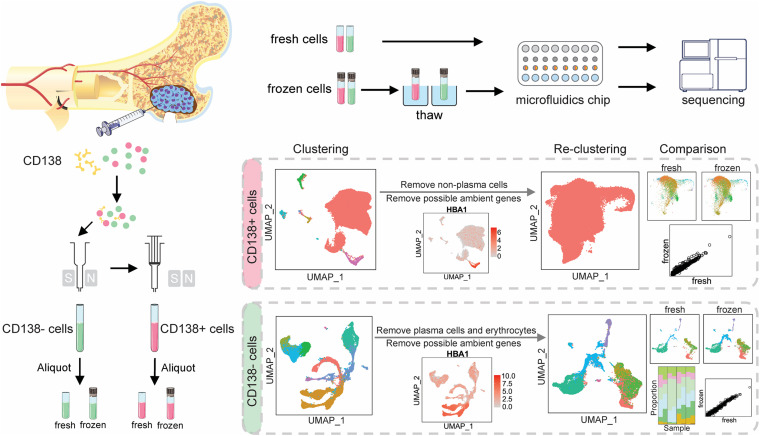
Overall schema for single-cell RNA sequencing and data analysis. Cells were isolated from bone marrow aspirates from six patients. Magnetic beads covalently coupled to CD138^–^ specific antibodies were used to sort cells into CD138^+^ and CD138^–^ populations. Both populations were then divided into two aliquots each; one was immediately processed for single cell RNA-sequencing and the other was cryopreserved and sequenced at a later date. CD138^+^ and CD138^−^ cells were analyzed separately to evaluate the impact of cryopreservation on cell population frequency and the transcriptome.

To investigate the impact of cryopreservation on myeloma cells and their microenvironment, cellular composition and gene expression profiles were compared between fresh vs. frozen/thawed cells that had been isolated from bone marrow aspirates from the same patient. Myeloma tumor cells were defined by expression of the plasma cell marker CD138 (CD138^+^), while CD138-negative (CD138^–^) cells defined the myeloma cell microenvironment, which is composed of osteoblasts and osteoclasts, as well as stromal, endothelial, and immune cells. CD138^+^ and CD138^–^ cells were sorted using magnetic beads covalently coupled to CD138^–^ specific antibodies and both cell populations were divided into two aliquots each; one was immediately processed for scRNA-seq (fresh) and the other was cryopreserved for sequencing at a later date (frozen) (Figure 1 and Supplementary Methods). The estimated numbers of cells for each sample from the scRNA-seq data are provided in Supplementary Table 1. The average numbers of cells per patient sample after performing quality control to remove low-quality cells (see section “Materials and Methods”) and incorrectly sorted cells were: 6,244 CD138^+^ fresh cells; 8,201 CD138^+^ frozen cells; 2,283 CD138^–^ fresh cells; and 5,397 CD138^–^ frozen cells.

### Cellular Composition Was Consistent Between Fresh and Frozen/Thawed CD138^+^ Cells

**FIGURE 2:**
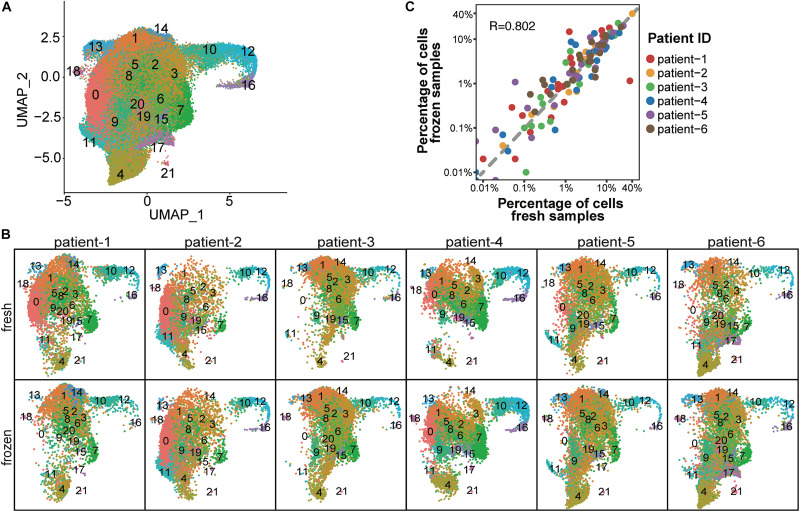
Cellular composition was consistent between fresh and frozen/thawed CD138^+^ cells. **(A)** UMAP plot of 37,463 fresh and 49,203 frozen CD138^+^ cells from 6 MM patient samples, where each cell is grouped into one of 22 clusters. Each cluster is distinguished by a different color and clusters are numbered from 0 to 21. **(B)** UMAP plots of cells identified in fresh and frozen samples from each patient. Cluster colors and numbers are the same as in **(A)**. **(C)** Scatter plot of the percentage of cells in each cluster in fresh samples (*x*-axis) vs. frozen samples (*y*-axis). Each dot represents one of 22 clusters; the color indicates a different patient.

To determine whether cryopreservation altered the cellular composition of CD138^+^ myeloma cells, we performed cell cluster analysis for each patient sample. To determine the number of cell clusters, we performed principal component analysis using the top 2,000 genes with the highest cell-to-cell variation in expression levels (Supplementary Methods). This analysis resulted in the assignment of a total of 37,463 fresh and 49,203 frozen CD138^+^ cells from six patients into 22 clusters (Figure 2A and Supplementary Table 1). The number of clusters did not differ between fresh and frozen samples from the same patient (Figure 2B), apart from four small clusters that contained less than 0.1% of the total number of cells (Supplementary Table 2). As demonstrated in Figure 2C, the percentage of cells in different clusters was consistent between the fresh and frozen samples (*R* = 0.802). These findings indicate that cryopreservation maintained the overall cellular composition in CD138^+^ cells.

### Gene Expression Profiles of Fresh and Frozen/Thawed CD138^+^ Cells Were Similar

**FIGURE 3:**
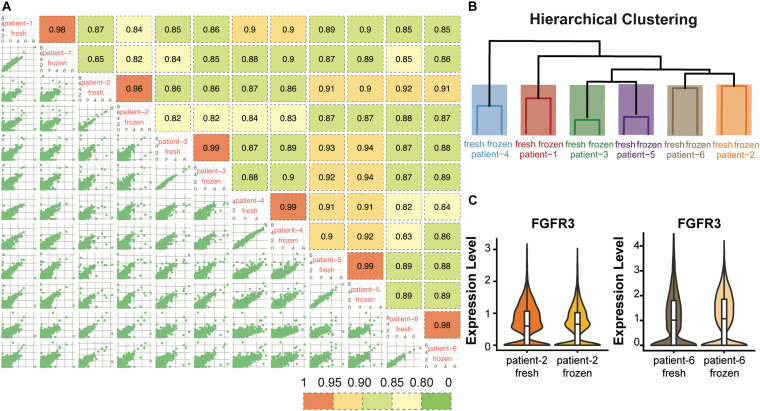
Gene expression profiles were consistent between fresh and frozen/thawed CD138^+^ cells. **(A)** Pairwise Pearson correlation matrix of the fresh and frozen samples from each patient. **(B)** Hierarchical clustering of the CD138^+^ fresh and frozen samples from each of 6 multiple myeloma patients. **(C)** Distribution and median RNA expression levels for *FGFR3* detected in fresh and frozen samples from patients 2 and 6, belonging to the t(4;14) multiple myeloma subgroup.

To evaluate whether cryopreservation maintained the gene expression levels in the CD138^+^ cells, we conducted pair-wise comparisons of the global gene expression levels for all fresh and frozen/thawed samples (Figure 3A). The global gene expression levels in each sample were calculated by averaging the normalized expression level of each gene in all cells (Supplementary Methods). Among all the comparisons, fresh and frozen samples from the same patient showed the highest correlation coefficient (*R* > 0.96). In addition, results from hierarchical clustering analysis showed that the fresh and frozen/thawed samples from the same patient were more similar to each other than they were to samples from different subjects (Figure 3B). This finding that inter-individual differences are preserved demonstrates that the freeze/thaw process had minimal effect on gene expression profiles.

We next asked whether the expression of key genes that define multiple myeloma cytogenetic subgroups were maintained following cryopreservation. Each of the six myeloma samples analyzed in this study belonged to one of the following three cytogenetic subgroups: hyperdiploid (HRD), translocation t(4;14), and translocation t(11;14). The chromosome 14 translocation subgroups are defined by high expression of key marker genes, including *CCND2, FGFR3*, and *WHSC1* for t(4;14) and *CCND1* and *SLC8A1* for t(11;14) (Stein et al., 2017; Supplementary Table 3). We found that the expression levels of these marker genes showed high consistency between fresh and frozen/thawed cells from the same patient. For example, the expression level of *FGFR3* showed highly similar expression profiles in fresh and frozen cells in both patients 2 and 6 (Figure 3C). Examples of the expression profiles of *CCND2* and *WHSC1* in patients 2 and 6, and of *CCND1* and *SLC8A1* in patient 3 are shown in Supplementary Figure 1. Together, these results demonstrate that cryopreservation had minimal effects on global gene expression levels or the expression levels of key genes defining subgroups with chromosome 14 translocations in CD138^+^ myeloma cells.

### Cell Populations in the Bone Marrow Microenvironment Were Consistent Between Fresh and Frozen/Thawed CD138^–^ Cells

**FIGURE 4:**
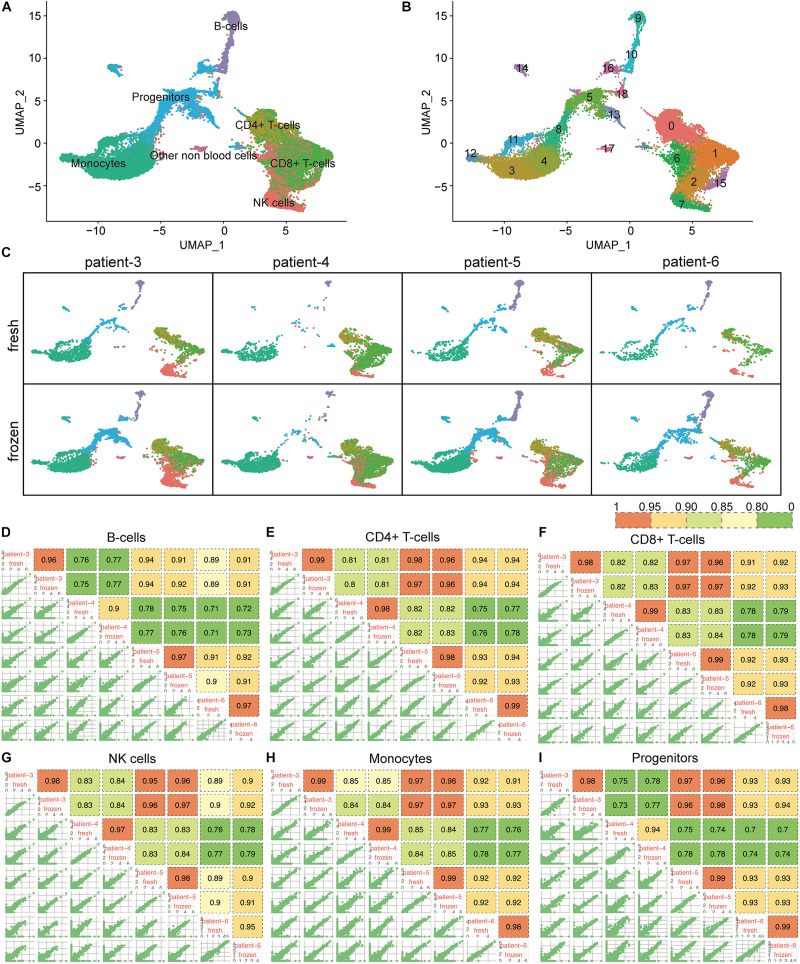
Cellular composition and gene expression profiles were consistent between fresh and frozen/thawed CD138^–^ cells. **(A)** UMAP plot of 9,131 fresh and 21,586 frozen/thawed CD138^–^ cells from 4 multiple myeloma patient samples. Each cell is grouped into one of seven cell types and each cell type is distinguished by a different color. **(B)** The same UMAP plot as **(A)** but each cell is grouped into one of 19 clusters. Each cluster is distinguished by a different color and clusters are numbered from 0 to 18. **(C)** UMAP plots of cells identified in fresh and frozen/thawed samples from each patient. Cluster colors are the same as in **(A)**. **(D–I)** Pairwise Pearson correlation matrix of the fresh and frozen/thawed CD138^–^ cells from each patient calculated for each major cell type separately.

Unlike CD138^+^ cells, which are mainly composed of plasma cells, the CD138^–^ cell population is heterogeneous, consisting of hematopoietic stem cells and blood cells in various stages of maturation. To determine whether cryopreservation might affect cell type composition in the CD138^–^ compartment, we analyzed scRNA-seq data from 9,131 fresh and 21,586 frozen/thawed CD138^–^ cells from four of the same six patients described above. The CD138^–^ cells were classified into 19 clusters representing seven different cell types (Figures 4A,B). We confirmed that every cell population identified in the fresh cells was also detected in the cryopreserved cells (Figure 4C). Similar to what was observed for the CD138^+^ cells, the number of clusters in fresh vs. frozen CD138^–^ samples did not differ, except for patient 5 where clusters containing non-blood cells (e.g., epithelial cells, fibroblasts, and keratinocytes) were not found in the fresh cells. While the total number of cells differed between fresh and frozen samples (Supplementary Table 1), our results indicate that all cell types were preserved in the frozen/thawed CD138^–^ samples.

### Gene Expression Profiles Were Consistent Between Fresh and Frozen/Thawed CD138^–^ Cells

We further examined whether cryopreservation altered the gene expression patterns in each of the major cell types composing the myeloma microenvironment (CD138^–^). Gene expression levels in the fresh and frozen cells from the same patient showed high correlation (*R* > 0.9) in all cell types (Figures 4D–I). Like the findings in the CD138^+^ cells, the correlation of gene expression profiles between fresh and frozen samples from the same patient were more similar to each other than to samples from different patients (Figures 4D–I). Based on these results, we conclude that cryopreservation also maintains gene expression patterns in CD138^–^ cell populations from bone marrow aspirates.

### Cryopreservation Affected the Relative Abundance of Cell Types in CD138^–^ Cells

**FIGURE 5:**
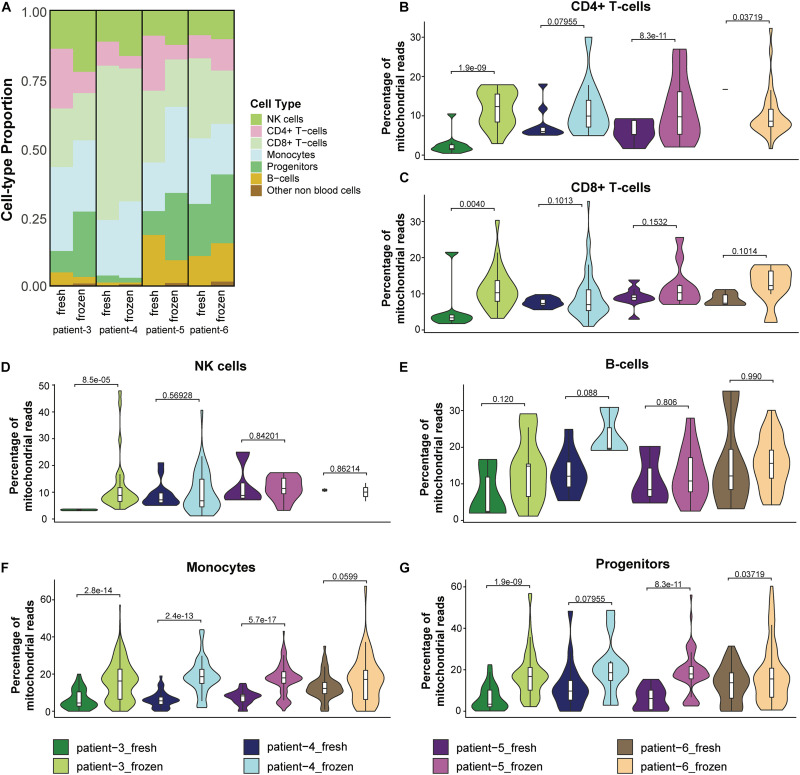
Cryopreservation affected the relative abundance of cell types in CD138^–^ cells. **(A)** Proportions of cell populations in fresh and frozen/thawed CD138^–^ samples across patients. **(B–G)** Distribution and median percentage of mitochondrial reads in filtered low-quality cells for each major cell type in each patient fresh and frozen/thawed sample. *T*-test was used to compare the percentage of mitochondrial reads among patients.

Because previous studies reported that cryopreservation of blood cells resulted in altered cell proportions of some immune cell types (Zheng et al., 2017; Bahsoun et al., 2019; Wohnhaas et al., 2019), we investigated whether the relative abundance of different immune cells was altered in cryopreserved CD138^–^ cells. We found that NK cells were more abundant in the frozen/thawed cells compared to the fresh samples; similarly, progenitor cells were also present at higher levels in frozen compared to fresh samples in three of the four patients (Figure 5A and Supplementary Table 2). In contrast, the proportion of B-cells and CD8^+^ T-cells were lower in the frozen/thawed cells compared with fresh cells and the proportion of CD4^+^ T-cells was lower in the frozen samples in 3 of 4 patients. Monocyte cell proportions also differed between fresh and frozen samples, although no consistent pattern was observed. Overall, the differences in cell proportions between fresh and frozen/thawed samples was on average less than 10% (Supplementary Table 2).

We next asked whether the observed differences in CD138^–^ cell proportions between fresh and frozen samples could be explained by differences in immune cell viability following thawing of the cryopreserved cells. One feature associated with cell stress and apoptotic cell death is a high number of mitochondrial transcripts (Ilicic et al., 2016). In our analysis, we considered cells having more than 15% mitochondrial reads as low-quality cells and removed them from the scRNA-seq data in the preprocessing quality control step. Therefore, we compared the percentage of mitochondrial reads in these low-quality cells and (Figures 5B–G) and the cells that passed the quality control filter (Supplementary Figures 2D–I) for each major cell type in the fresh and frozen/thawed samples from each patient. *T*-test was used to measure the statistical difference of the percentage of mitochondrial reads in the fresh and frozen/thawed samples from the same patient. We found the percentage of mitochondrial reads for each cell type was higher in the cryopreserved cells (Figures 5B–G), indicating that the freeze/thaw process likely generates a stress response in all cell types. These data suggest that the observed changes in cell type proportions (e.g., lower T-cells and higher progenitor cells) in the cryopreserved cells reflect the lower viability of T-cells relative to other cell types following freeze/thaw. Consequently, while cryopreservation maintains the cell populations present in fresh CD138^–^ cells, the freeze/thaw process can result in small changes to the relative abundance of each cell type.

## Discussion

In this study, we evaluated the impact of cryopreservation on both multiple myeloma cells isolated from bone marrow aspirates and their microenvironment using scRNA-seq. The major conclusions of the current study are that cryopreservation of bone marrow aspirates from patients with multiple myeloma had minimal effect on the number of cell clusters, the proportion of major cell types, or gene expression patterns compared to freshly processed cells for both the CD138^+^ plasma cells as well as the more heterogeneous CD138^–^ cell population.

Our findings are consistent with those of others who have performed scRNA-seq on both fresh and cryopreserved cells and tissues (Guillaumet-Adkins et al., 2017; Zheng et al., 2017; Wohnhaas et al., 2019). For example, Guillaumet et al., was the first to report that DMSO cryopreservation maintained gene expression profiles in PBMC’s and also in mouse colon tissue and orthotopic tumor models. The study by Zheng and colleagues that first described the droplet-based scRNA-seq technology used in our study, also compared fresh and DMSO cryopreserved PBMCs from the same donor. This group reported a two-fold increase in 57 genes in the frozen/thawed cells with no differences in cell-type proportions (Zheng et al., 2017). In addition, Wohnhaas et al., found minimal gene expression changes when comparing fresh to DMSO cryopreserved cell lines, monocyte-derived macrophages, and primary immune cells enriched from rat liver, although they noted increased expression of *FOS*, *FOSB*, and *JUN* in the frozen/thawed cells (Wohnhaas et al., 2019). They also reported that gene expression profiles from fresh and frozen/thawed primary immune cells from the same animal were more similar to each other than to samples across animals. Similar to our findings, Wohnhaas et al., reported that while DMSO cryopreservation maintained all of the immune cell populations and the relative abundance per cell population found in the fresh samples, some variability was observed across different animals, most notably in the proportions of NK cells and dendritic cells. Therefore, while DMSO cryopreservation maintains highly similar cell proportions and gene expression profiles, small perturbations cannot be avoided completely and comparisons across fresh and cryopreserved cells are not recommended.

Although cryopreservation had minimal effect on the gene expression patterns compared to freshly processed cells, we noticed higher background contamination levels from transcripts from damaged or dead cells. The presence of these transcripts is a common problem in droplet-based scRNA-sequencing technology (Chesi and Bergsagel, 2011; Wohnhaas et al., 2019), which can bias the analysis. Although we performed a red blood cell lysis step, highly expressed red blood cell marker genes, *HBB, HBA1*, and *HBA2*, were still observed in all non-erythrocyte cell types, indicating the presence of cross-cell contamination (Supplementary Figure S3 and Supplementary Methods). To minimize this bias in our analysis, we performed a gene selection step (Supplementary Methods); however, some bias will remain that needs to be considered.

Our work shows that cryopreservation of cells from bone marrow aspirates of multiple myeloma patients maintained consistent gene expression profiles across all cell types, as well as preserved the overall cell type proportions compared with freshly prepared cells. This finding provides experimental support for the use of cryopreservation protocols in scRNA-seq gene expression studies of multiple myeloma cells, which will greatly facilitate the use this technology in longitudinal clinical studies of this disease.

## Materials and Methods

### Patient Samples

Bone marrow aspirates were obtained from patients enrolled in The Indiana Myeloma Registry (Clinicaltrials.gov ID: NCT03616483). This is an IRB-approved prospective, non-interventional study collecting comprehensive clinical, genomic, demographic, social, environmental and quality of life data from subjects with plasma cell dyscrasias.

### Cell Preparation

Human bone marrow mononuclear cells (BM MNCs) were collected in heparin tubes from multiple myeloma patients and were isolated by Ficoll-Paque PLUS (General Electric). Briefly, bone marrow aspirates were diluted twofold with phosphate-buffered saline (PBS) and layered over Ficoll-Paque PLUS in a 15 ml tube and centrifuged at 400 × *g* for 30 min at room temperature with the brake off. After removing the top layer of plasma, BM MNCs were collected and diluted with 3 volumes of PBS buffer, and washed once at room temperature. The red blood cells (RBC) were removed by addition of 7 ml RBC lysis buffer to the cell pellet and incubation at room temperature for 2 min, followed by centrifugation at 1,400 rpm for 5 min and one time wash with cold PBS. CD138^+^ and CD138^–^ cells were then sorted using CD138 Microbeads (Miltenyi Biotec), based on the standard MACS separation protocol (Miltenyi Biotec).

### Cell Cryopreservation

Freezing medium (90% FCS, 10% DMSO) was added to 5 × 10^5^ to 1 × 10^6^ cells/mL and cells were transferred to a cryogenic vial. The vials were put into a Mr. Frosty freezing container and stored at −80°C overnight. Vials were stored in liquid nitrogen until use.

### Cell Thawing

Frozen cells were thawed slowly according to the Demonstrated Protocol for Fresh Frozen Human Mouse Cell line Mix, CG00014 RevF (10X Genomics, Pleasanton, CA). Briefly, a vial of cells was removed from liquid nitrogen storage and thawed in a water bath at 37°C until only a small ice crystal remained. Pre-warmed RPMI-1640 media containing 10% FBS (RPMI + FBS) was added to the cryovial and gently mixed with a wide-bore pipette tip. Cells were transferred to a 50 ml conical tube, followed by sequential additions of 2, 4, 8, and 16 ml of RPMI + FBS at a rate of 1 ml per 5 s. Cells were washed 3 times by pelleting cells at 300 × *g* for 5 min at room temperature and gently resuspending in 9 ml of RPMI + FBS. If cell aggregations were observed, the cell suspensions were filtered through a 40 μm Flowmi Cell Strainer (Bel-Art SP Scienceware, Wayne, NJ). Cell concentration and viability were determined, and cell concentration was adjusted to approximately 1,000 cells per μl. According to 10X Genomic protocol, the targeted cells per sample is 10,000 cells per μl for cell recovery.

### Sequencing Library Construction

Single-cell 3′ gene expression libraries were prepared for each sample targeting10,000 cells for cell recovery using the Chromium Single Cell 3′ Reagent Kits v2 and the Chromium single-cell system (10X Genomics, Inc). The resulting library was sequenced using a custom program for 26 bp plus 98 bp paired-end sequencing on a NovaSeq 6000 sequencer (Illumina, Inc, San Diego, CA). Approximately 50,000 reads per cell were generated.

### Data Analysis Procedure

Two rounds of data processing were performed using Seurat V3 (Stuart et al., 2019) and *SingleR*. In the first round, low-quality droplets were removed, and cell types were annotated. Low quality droplets were defined as those containing < 600 total unique molecular identifiers, or the percentage of mitochondrial reads were > 15% (Supplementary Table 1). Following cell-type annotation, we identified contaminating cells (i.e., non-plasma cells in CD138^+^ cells, and plasma cells and erythrocytes in CD138^–^ cells) and identified genes highly expressed in these cells using *FindMarkers* function with default parameters in Seurat. In the second round, the incorrect cells were removed before performing the standard processing procedure with Seurat to cluster cells. *SingleR* was used for cell type annotation. The detailed steps of data processing can be found in the Supplementary Methods.

### Consistency of Gene Expression Profile

Consistency of gene expression between fresh and preserved cells was calculated using Pearson correlation test function in R 4.0. To assess the overall consistency, we calculated the average gene expression profiles of each patient per cell type across samples for CD138^+^ cells and CD138^–^ cells separately. These average gene expression profiles were calculated for the entire gene set using the *AverageExpression* function in Seurat V3 and log2 transformed.

## Data Availability Statement

Sequence data are available in GEO with accession no. GSE161722. The interactive visualization inference was set up with UCSC Cell Browser: https://clark.ccbb.iupui.edu/PHI_MM_Pilot/.

## Ethics Statement

The studies involving human participants were reviewed and approved by the Turik, Michael A The Indiana University Institutional Review Board. The patients/participants provided their written informed consent to participate in this study.

## Author Contributions

DC: bioinformatics data analyses, and manuscript writing. MA: study design and sample collection. JR: manuscript editing. MC and LW: process the samples including most the cell biology components. PM and XX: conducted single cell analysis. HG: conducted single cell study design and data processing. KH: provide scientific input for the study design. RA: sample collection, provide scientific input for the study design. BW: provide scientific input for the study design. YL: study supervision, data interpretation and manuscript editing. All authors contributed to the article and approved the submitted version.

## Conflict of Interest

The authors declare that the research was conducted in the absence of any commercial or financial relationships that could be construed as a potential conflict of interest.

## Supplementary Material

Supplementary Figure 1 Comparison of marker gene expression levels of multiple myeloma patients between fresh and cryopreserved CD138^+^ cell. **(A,B)** Expression profiles of marker genes *CCND2* and *WHSC1* for patient 2 in t(4:14) multiple myeloma subgroup. **(C,D)** Expression profiles of marker genes *CCND1* and *SLC8A1* for patient 3 in t(11:14) multiple myeloma subgroup. **(E)** Expression profiles of marker gene *CCND2* for patient 6 in t(4:14) multiple myeloma subgroup.

Supplementary Figure 2 Percentage of reads mapped to the mitochondrial genes. **(A)** Percentage of reads mapped to mitochondrial genes of the original CD138^+^ cells across patients. **(B)** Percentage of reads mapped to mitochondrial genes of the original CD138^–^ cells across patients. **(C)** Pairwise Pearson correlation matrix of the fresh and frozen samples from each patient for CD138^–^ cells. **(D–I)** Distribution and median percentage of mitochondrial reads in good quality cells for each major cell type in each patient fresh and frozen/thawed sample. *T*-test was used to compare the percentage of mitochondrial reads among patients.

Supplementary Figure 3 Expression distribution of genes with higher background contamination levels. **(A,B)** Expression distribution of *HBB*, *HBA1*, and *HBA2* for CD138^+^ **(A)** and CD138^–^ cells **(B)**, which showed high expression levels in erythrocytes and were also found in all other cell types.

Supplementary Table 1 Distribution of the number of cells before and after quality control, gene selection, and cell type removal. Tab 1, CD138^+^ cells; Tab 2, CD138^–^ cells.

Supplementary Table 2 Number of cells in each cluster. Tab 1, CD138^+^ cells; Tab 2, CD138^–^ cells.

Supplementary Table 3 Key genes of each multiple myeloma subgroup.

Supplementary Table 4 Cell load, processing data and viability.

Supplementary Table 5 Genes removed in the gene selection step. Tab 1, CD138^+^ cells; Tab 2, CD138^–^ cells.
