# Supplementary figures and images for: Cryopreservation Preserves Cell-Type Composition and Gene Expression Profiles in Bone Marrow Aspirates From Multiple Myeloma Patients
Source: Front Genet. 2021 Apr 21;12:663487. doi: 10.3389/fgene.2021.663487 (PMC8099152; doi:10.3389/fgene.2021.663487)

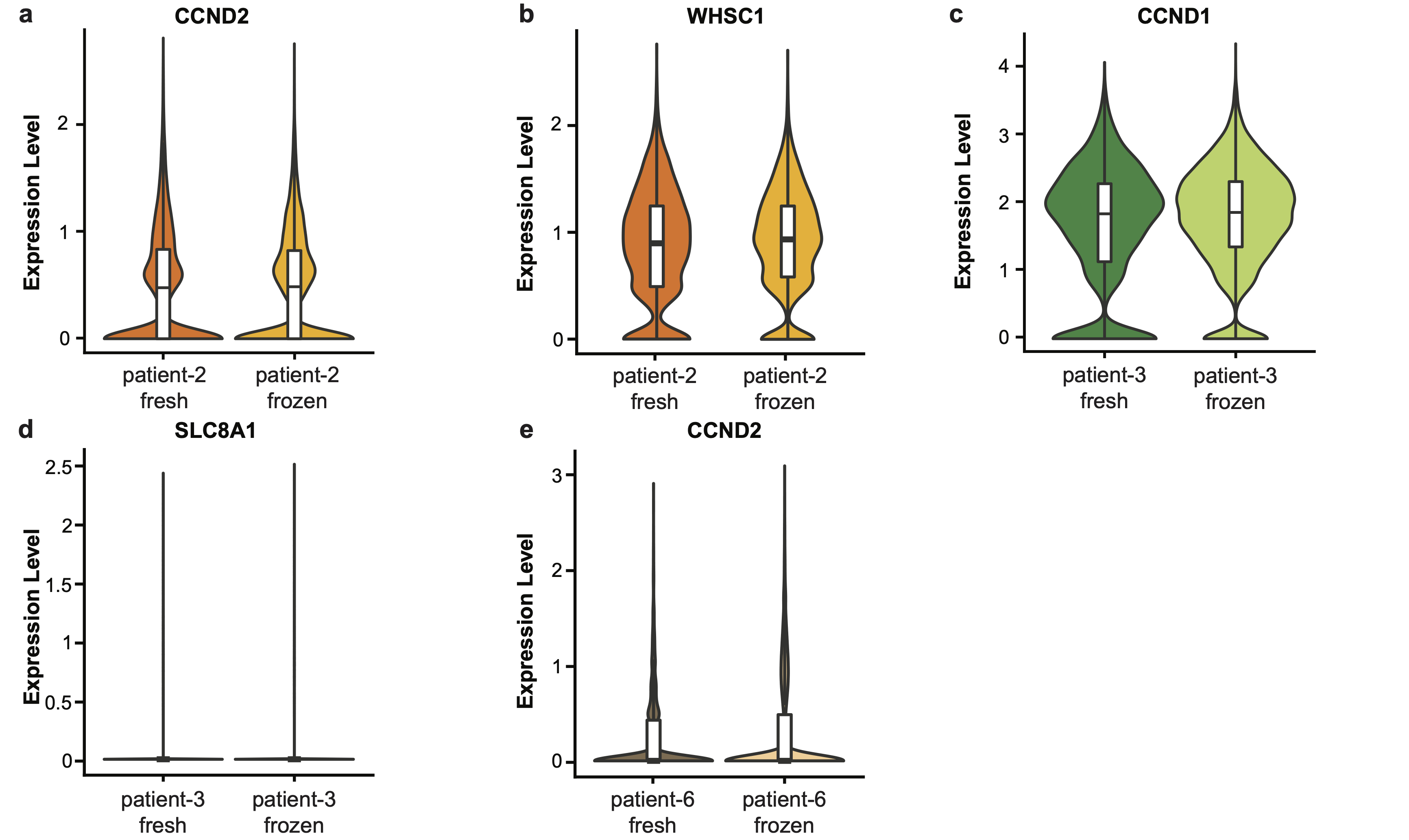

Supplement: Supplementary Figure 1 — Comparison of marker gene expression levels of multiple myeloma patients between fresh and cryopreserved CD138+ cell. (A,B) Expression profiles of marker genes CCND2 and WHSC1 for patient 2 in t(4:14) multiple myeloma subgroup. (C,D) Expression profiles of marker genes CCND1 and SLC8A1 for patient 3 in t(11:14) multiple myeloma subgroup. (E) Expression profiles of marker gene CCND2 for patient 6 in t(4:14) multiple myeloma subgroup. [file Image_1.JPEG]

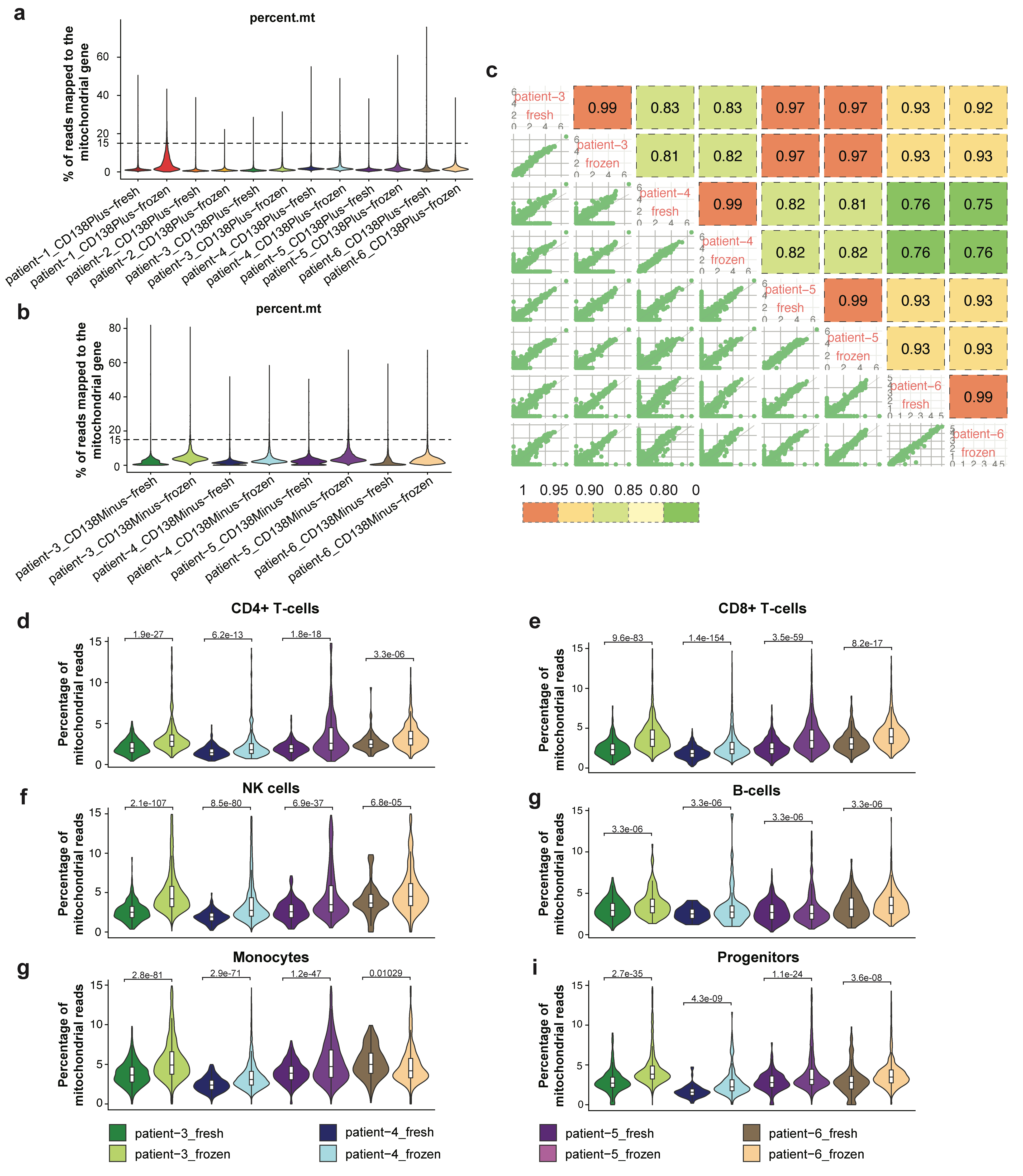

Supplement: Supplementary Figure 2 — Percentage of reads mapped to the mitochondrial genes. (A) Percentage of reads mapped to mitochondrial genes of the original CD138+ cells across patients. (B) Percentage of reads mapped to mitochondrial genes of the original CD138– cells across patients. (C) Pairwise Pearson correlation matrix of the fresh and frozen samples from each patient for CD138– cells. (D–I) Distribution and median percentage of mitochondrial reads in good quality cells for each major cell type in each patient fresh and frozen/thawed sample. T-test was used to compare the percentage of mitochondrial reads among patients. [file Image_2.JPEG]

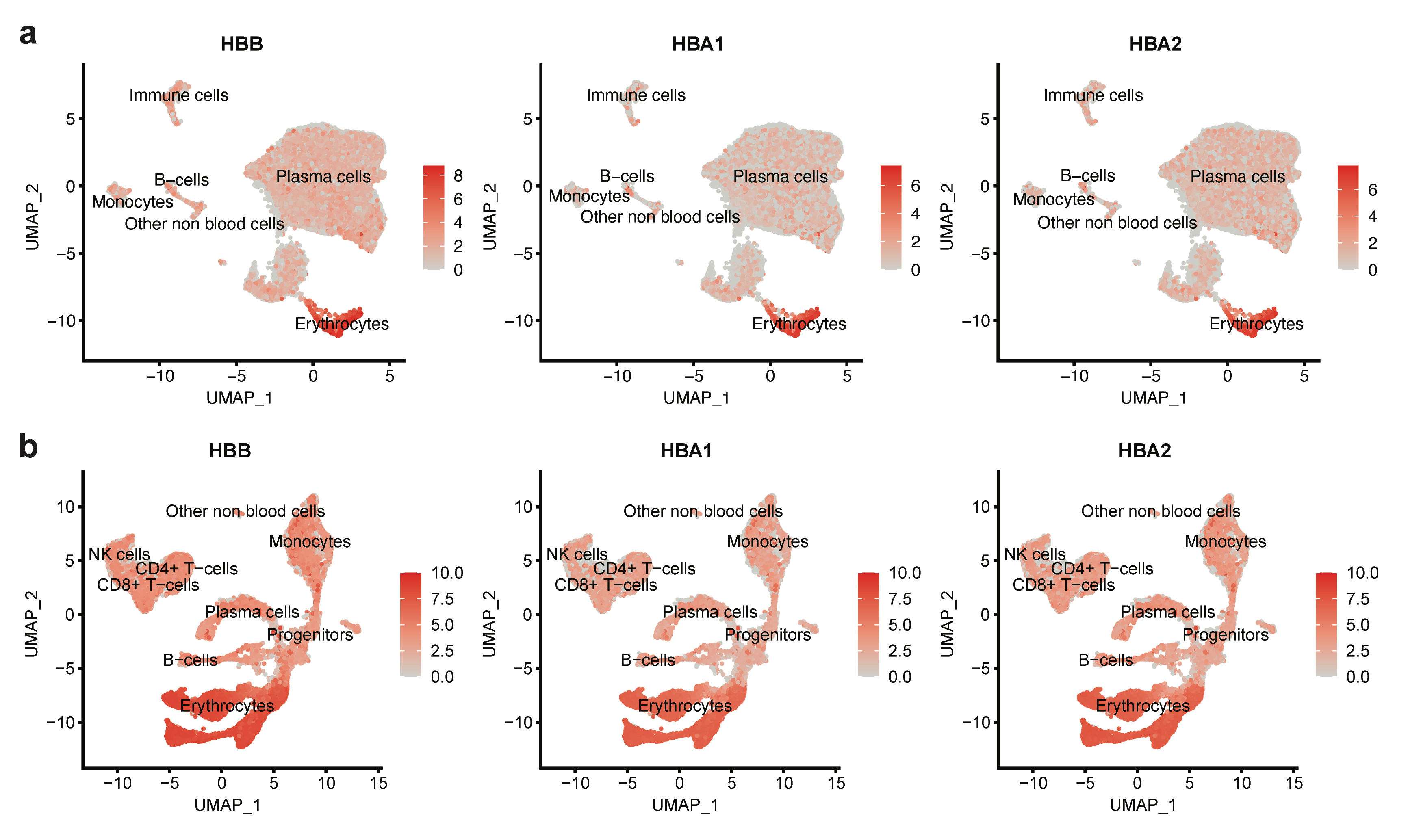

Supplement: Supplementary Figure 3 — Expression distribution of genes with higher background contamination levels. (A,B) Expression distribution of HBB, HBA1, and HBA2 for CD138+ (A) and CD138– cells (B), which showed high expression levels in erythrocytes and were also found in all other cell types. [file Image_3.JPEG]
