# Supplementary material for: Cryopreservation Preserves Cell-Type Composition and Gene Expression Profiles in Bone Marrow Aspirates From Multiple Myeloma Patients
Source: Front Genet. 2021 Apr 21;12:663487. doi: 10.3389/fgene.2021.663487 (PMC8099152; doi:10.3389/fgene.2021.663487)
Supplement: Supplementary file 9 [file Table_6.DOCX]

**Supplementary Materials and Methods**

**Sequencing library construction.** Single-cell 3’ gene expression libraries were prepared using the Chromium Single Cell 3’ Reagent Kits v2 and the Chromium single-cell system (10X Genomics, Inc). We targeted at 10,000 cells per sample for cell recovery for GEM partition (emulsion), according to 10X Genomics protocol. The cell number and viability were counted using hemocytometer. This step is different from bulk RNA-seq in which all cells in a given sample were lysed in bulk and total RNA extracted, with no requirement for cell counting. Although the target cell numbers could be different across samples, it shouldn’t be too far from the cell number per sample from CellRanger. The resulting library was sequenced using a custom program for 26 bp plus 98 bp paired-end sequencing on a NovaSeq 6000 sequencer (Illumina, Inc, San Diego, CA). Approximately 50,000 reads per cell were generated.

**Preprocessing of single-cell data**

CellRanger (v3.1.0, 10X Genomics) was used to demultiplex raw base call files, perform barcode counting, unique molecular identifier (UMI) counting, and align reads to the human reference genome (10X Cellranger reference GRCh38 v3.0.0). Cells with very few genes detected or with aberrantly high gene counts were filtered. The thresholds used for this quality control are provided in Table S1. Cells in the process of dying were identified as having more than 15% of reads mapped to mitochondrial genes and were also removed (Fig S2a,b). Gene expression measurements for each cell were calculated as the log-transformation of the number of reads mapped to each gene normalized by the total expression of all genes, multiplied by the scale factor 10,000.

**Cell clustering**

The top 2,000 genes with highest cell-to-cell variation in expression levels were selected as the highly variable features and used in the downstream dimensional reduction analysis. The expression of these 2,000 genes was z-normalized, making the mean expression across cells equal to 0 and scaling the expression to make the variance across cells equal to 1, which gives these genes equal weight and avoids possible domination of highly expressed genes. Principal component analysis was performed on the processed data and the dimensionality of the dataset was determined with a Jackstraw plot, identifying significant PCs with strong enrichment of low p-value features (77 for CD138+ cells and 55 for CD138- cells). K-nearest neighbor clustering with resolution 1.2 for CD138+ cells and 0.8 for CD138- cells was performed on the number of PCs we determined for the resulting data to cluster the cells. Cells were visualized by the non-linear dimensional reduction technique using uniform manifold approximation and projection (UMAP).

**Cell type annotation and incorrect cell types removal**

Cell type annotation was performed using SingleR [1]. We selected the reference dataset BlueprintEncode, which includes the normalized expression values and cell type labels based on bulk RNA-seq from Blueprint (Martens and Stunnenberg 2013) and ENCODE Project Consortium 2012. For CD138+ fractions, any non-plasma cell clusters were removed. For the CD138- fractions, only non-plasma and non-erythroid clusters were analyzed.

**Gene selection**

The droplet-based approach is commonly used in current single-cell sequencing experiments. However, mRNA that has been released from dead cells can also be incorporated into droplets and become barcoded [2]. Although this background contamination is undetectable in many cases, highly expressed genes may lead to misleading biological interpretation and bias in the downstream analysis [3, 4]. To detect the effect of these genes on our analysis, we assessed the expression distribution of typical genes, *HBB*, *HBA1*, and *HBA2* (FigS3). We processed the data sequenced from CD138+ cells and CD138- cells with the standard Seurat processing workflow without any cell type selection and used UMAP to visualize the cell clusters and the gene expression level distribution. In both CD138+ and CD138- samples, *HBB*, *HBA1*, and *HBA2* were highly expressed not only in the erythrocyte cluster, but also in all other cell types, indicating a cross-cell contamination effect (FigS3). Therefore, an additional quality control step was implemented by selecting genes with ultra-high expression in the excluded cells (i.e., non-plasma cells for CD138+ cells, and plasma cells and erythrocytes for CD138- cells), in the downstream analysis to remove this effect. The genes removed from the analysis are provided in Table S6. In CD138+ samples, 229 genes that were highly expressed in the non-plasma cells were removed. In CD138- samples, 377 genes that were highly expressed in plasma cells and erythrocytes were removed.

**References**

1 Aran D, Looney AP, Liu L, Wu E, Fong V, Hsu A *et al*. Reference-based analysis of lung single-cell sequencing reveals a transitional profibrotic macrophage. *Nat Immunol* 2019; 20: 163-172.

2 Yang S, Corbett SE, Koga Y, Wang Z, Johnson WE, Yajima M *et al*. Decontamination of ambient RNA in single-cell RNA-seq with DecontX. *Genome Biol* 2020; 21: 57.

3 Young MDB, S. SoupX removes ambient RNA contamination from droplet based single cell RNA sequencing data. *bioRxiv* 2018.

4 Wohnhaas CT, Leparc GG, Fernandez-Albert F, Kind D, Gantner F, Viollet C *et al*. DMSO cryopreservation is the method of choice to preserve cells for droplet-based single-cell RNA sequencing. *Sci Rep* 2019; 9: 10699.
